# Effect of dupilumab on otitis media associated with eosinophilic chronic rhinosinusitis

**DOI:** 10.1016/j.bjorl.2024.101555

**Published:** 2025-01-03

**Authors:** Seiichiro Kamimura, Keisuke Ishitani, Ryota Morozumi, Eiji Kondo, Takahiro Azuma, Go Sato, Yoshiaki Kitamura

**Affiliations:** Tokushima University Graduate School, Institute of Biomedical Sciences, Department of Otolaryngology-Head and Neck Surgery, Tokushima, Japan

**Keywords:** Eosinophilic otitis media, Eosinophilic chronic rhinosinusitis, Dupilumab, Biologics

## Abstract

**Objective:**

Eosinophilic Otitis Media (EOM) is an intractable disease caused by type 2 inflammation, such as Eosinophilic Chronic Rhinosinusitis (ECRS) and bronchial asthma. Biologics have recently been used to treat ECRS and bronchial asthma. Biologics are not indicated for EOM; however, because approximately 10% of ECRS cases has concomitant EOM, concomitant EOM improvement has been observed when dupilumab is administered for ECRS. Therefore, we aimed to investigate the current status of EOM treatment and clarify the effect of dupilumab on EOM.

**Method:**

This retrospective study included 25 patients attending our hospital. The disease status of patients with EOM not treated with dupilumab (control) and those treated with dupilumab at the time of evaluation were compared. The EOM disease status was compared using the EOM severity score, temporal bone shadow on Computed Tomography (CT), and standard pure tone audiometry.

**Result:**

Twenty-four of the 25 patients with EOM had concomitant ECRS, and 12 were administered with dupilumab. The severity score of EOM, temporal bone CT score, and air conductance hearing were significantly improved after dupilumab administration in 12 patients with EOM. Comparison of EOM patients without dupilumab (control) and those treated with dupilumab at the time of evaluation revealed that the severity score of EOM, temporal bone CT score, and air conduction hearing were significantly better in patients treated with dupilumab.

**Conclusion:**

Our study demonstrated that dupilumab was administered to approximately half of the patients with EOM, suggesting that the treatment of EOM has undergone significant changes due to dupilumab administered to ECRS. Our study also suggests that dupilumab is highly effective in patients with EOMs.

**Level of evidence:**

4.

## Highlights

• Dupilumab improved EOM severity and CT scores in patients with concomitant ECRS.
• Air-conductive hearing in EOM patients improved significantly with dupilumab.
• Dupilumab reduced steroid use in EOM patients, suggesting long-term benefits.
• EOM treatment has shifted significantly with dupilumab use for concomitant ECRS.
• Dupilumab is highly effective in treating EOM, improving clinical outcomes.

## Introduction

Eosinophilic Otitis Media (EOM) is refractory otitis media with eosinophilic infiltration of the middle ear mucosa and effusion containing eosinophils that accumulate in the middle ear.^1^ Patients with EOM may experience a sense of ear fullness and hearing loss due to middle ear effusion, and in cases of perforated tympanic membranes, very viscous ear discharge may be present. Initially, hearing loss due to EOM is a reversible conductive hearing loss; however, as it progresses, it can lead to irreversible sensorineural hearing loss and even deafness in severe cases, greatly reducing the quality of life of patients.^2^, ^3^, ^4^ EOM is treated with topical or oral corticosteroids but is generally refractory and recurrent.^1^

EOM is a type 2 inflammation characterized by eosinophil infiltration of the middle ear mucosa, similar to Eosinophilic Chronic Rhinosinusitis (ECRS), which is characterized by eosinophil infiltration of the nasal mucosa.^1^ Type 2 cytokines such as IL-4, IL-5, and IL-13 promote eosinophil differentiation and migration into peripheral tissues and affect airway inflammation caused by eosinophils and type 2 cytokines. In recent years, many biologics have been developed to treat type 2 inflammation. Dupilumab, an antibody against the receptors for IL-4 and IL-13, has been available in Japan for the treatment of Chronic Sinusitis with Nasal Polyps (CRSwNP) since March 2020. Since then, it has mainly been administered for ECRS, a typical CRSwNP, with optimal therapeutic results.^5^, ^6^ Although dupilumab is not directly indicated for EOM because approximately 10% of ECRS cases has concomitant EOM,^1^ cases of concomitant EOM improvement have been observed when dupilumab is administered for ECRS. Thus, to clarify the effect of dupilumab on EOM, we compared the status of EOM before and after dupilumab administration in patients with ECRS and concomitant EOM treated with dupilumab. Furthermore, we compared the disease status of patients with EOMs who were administered dupilumab with those who were not.

## Methods

A retrospective study was conducted on 25 patients with EOM attending the Department of Otolaryngology-Head and Neck Surgery at Tokushima University Hospital as of March 31, 2024. In patients with EOMs treated with dupilumab for concomitant ECRS, the EOM status was compared before and 3-months after dupilumab administration. The status of EOM at the time of evaluation was also compared between patients with EOMs treated with and without dupilumab. The blood eosinophil count was aggregated and compared in the absence of dupilumab or oral corticosteroid administration. EOM were diagnosed according to the diagnostic criteria described by Iino et al.^7^ We also included suspected EOM cases with middle ear effusion in which eosinophils were not proven before dupilumab administration; however, EOM associated with ECRS and bronchial asthma was strongly suspected clinically. The standard treatment for EOM consisted of intratympanic injections of triamcinolone. For refractory cases, oral corticosteroids were administered.

The severity of EOM and temporal bone shadows on CT was evaluated using a score devised by Iino et al.^8^, ^9^ Hearing was evaluated by standard pure tone audiometry (Rion AA-H1). All ECRS patients were cases with a definitive histopathological diagnosis, confirmed by the presence of 70 or more eosinophils per High-Power Field (HPF) in nasal polyps, scoring 11 points or higher on the Japanese Epidemiological Survey of Refractory Eosinophilic Chronic Rhinosinusitis (JESREC) score^10^ (Table 1). Dupilumab for ECRS was administered to patients who met the administration criteria (Table 2). All patients treated with dupilumab were administered dupilumab for at least 3 months. Statistical analysis was performed using EZR software (Saitama Medical Center, Jichi Medical University, Saitama, Japan). Comparisons were made before and after dupilumab administration using Wilcoxon signed-rank test and between dupilumab-treated cases and controls using Mann-Whitney *U* test and χ^2^ test. This study was approved by the Ethics Committee of the University of Tokushima University (4060-1).

**Table 1:** JESREC score criteria for the diagnosis of eosinophilic chronic rhinosinusitis.

Factor	Score
Disease side: both sides	3
Nasal polyp	2
CT shadow: ethmoid ≥ maxillary	2
Eosinophils of peripheral blood	
2% < ‒ ≤ 5%	4
5% < ‒ ≤ 10%	8
10% <	10

Diagnosis	JESREC score
ECRS	≥ 11
Non-ECRS	≤ 10

**Table 2:** The indications for dupilumab for chronic rhinosinusitis with nasal polyps in Japan.

A definitive diagnosis of chronic rhinosinusitis has been made, and the patient meets one of the following criteria:
a. “Has a history of systemic steroid treatment for chronic rhinosinusitis with nasal polyps within the past two years”.
b. “Has undergone surgical treatment for chronic rhinosinusitis with nasal polyps”.
c. “Is contraindicated for systemic steroid use or demonstrates intolerance to it”.
In addition, despite receiving existing treatments, the patient satisfies all of the following three conditions.
1) Endoscopic examination reveals a nasal polyp score (0–4-points per side) of at least 2-points in each nasal cavity, with a total score of 5-points or higher for both sides combined.
2) The nasal obstruction severity score (range: 0–3, where 0 = no symptoms, 1 = mild symptoms, 2 = moderate symptoms, and 3 = severe symptoms) is 2 or higher and has persisted for at least 8-weeks.
3) Symptoms such as olfactory dysfunction or nasal discharge (anterior/posterior rhinorrhea) have persisted for at least 8-weeks.

## Results

The mean age of the 25 patients was 57.4 ± 12.2 years, with eight males and 17 females (Table 3). Twenty patients were definitive cases of EOM, and five were suspected cases. Twenty-four patients with EOM also underwent concomitant ECRS. Bronchial asthma was present in all patients. Among the 25 patients, 12 were administered dupilumab for concomitant ECRS. The mean JESREC score in these 12 patients was 15.7 ± 1.8. There were four cases of regular oral Prednisolone (PSL) use and two cases of intermittent PSL use in the past 3-months.

**Table 3:** Clinical profile of the patients.

	Total	Control	Dupilumab	*p* value
n	25	13	12	‒
Age ± SD (Median)	57.4 ± 12.2 (58)	60 ± 13.8 (63)	54.5 ± 10.0 (57)	0.26
Sex Men:Female	8:17	4:9	4:8	0.89
Blood eosinophil count (%)	14.2 ± 6.8	14.9 ± 4.5	13.4 ± 8.9	0.6
Blood eosinophil count	978 ± 594	1027 ± 333	925 ± 802	0.68
EOM definitive (%)	20 (80)	12 (92)	8 (67)	0.11

**Complications**				
ECRS (%)	24 (96)	12(92)	12 (100)	0.32
Bronchial Asthma (%)	25 (100)	13 (100)	12 (100)	–

**Treatment**				
Dupilumab (%)	12 (48)	0 (0)	12 (100)	–
Regular use of oral PSL (%)	4 (16)	4 (31)	0 (0)	0.036
Oral PSL intermittent use (%) (past 3-months)	2 (8)	2 (15)	0 (0)	0.16

**Fig. 1:**
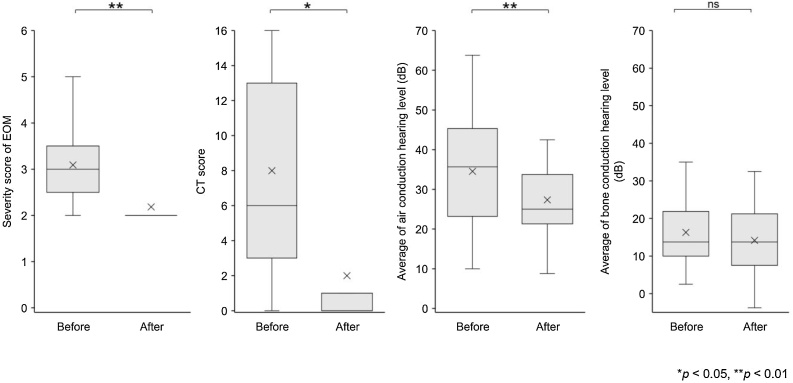
Comparison between before and 3-months after dupilumab administration. *p < 0.05, **p < 0.01, ns not significant; Wilcoxon signed-rank test.

**Fig. 2:**
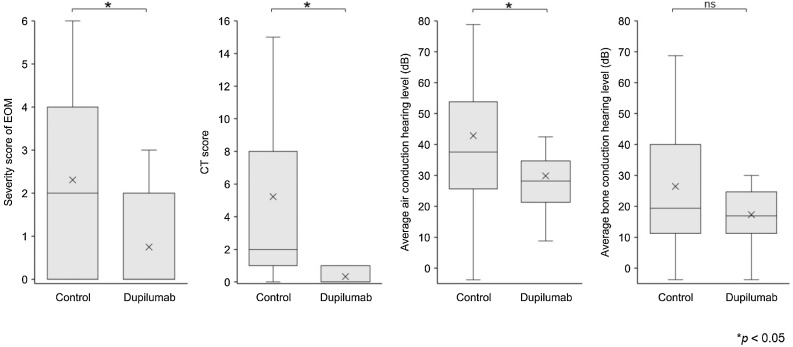
Comparison between patients without dupilumab (control) and with dupilumab (dupilumab). *p < 0.05, ns not significant; Mann–Whitney *U* test.

Comparing the 12 patients treated with dupilumab before and after 3-months of continuous treatment, the EOM severity score decreased from 3.1 ± 0.9 to 2.2 ± 0.6 (*p* = 0.002), temporal bone CT score decreased from 8.0 ± 6.4 to 1.8 ± 4.6 (*p* = 0.016), standard pure tone audiometry results for air conductive hearing (quadrant method) improved from 34.5 ± 14.9 to 27.2 ± 13.5 (*p* = 0.005), and bone conductive hearing (quadrant method) did not significantly improve (16.3 ± 9.4 to 13.5 ± 10.2) (*p* = 0.18) (Fig. 1). None of the patients in the dupilumab group required regular or intermittent PSL, whereas four patients in the control group required regular PSL and two patients required intermittent PSL (Table 1). Dupilumab was administered once every 2-weeks in all patients treated with dupilumab for a mean of 106.1 ± 44.2 weeks at the time of evaluation. EOM severity scores at the time of evaluation of the control group and dupilumab group were 2.3 ± 2.3 and 0.75 ± 1.1 (*p* = 0.04), temporal bone CT scores were 5.2 ± 5.7 and 0.3 ± 0.5 (*p* = 0.007), standard pure tone audiometric results for air conductive hearing (quadrant method) were 42.9 ± 26.4 and 29.2 ± 16.8 (*p* = 0.049), and the scores for bone conductive hearing (quadrant method) were 26.4 ± 22.3 and 17.1 ± 10.9 (*p* = 0.09) (Fig. 2).

## Discussion

We conducted a retrospective study of all patients with EOM who visited our hospital. The 25 patients with EOM were predominantly female with a mean age of 50 s, and these results were similar to a previous report.^7^ Interestingly, ECRS is more common among males while EOM is more common among females.^10^ It has been suggested that patulous eustachian tubes exist in the background of patients with EOM and that airway inflammation may spill over into the middle ear via the eustachian tube,^11^ and patulous eustachian tubes are more common in females which may be one reason why EOM is more common in females.^12^, ^13^, ^14^

Approximately 10% of ECRS cases are associated with EOM, and conversely, 70%–80% of EOM are associated with ECRS,^7^, ^12^, ^15^ thus, EOM is less common than ECRS. However, the number of patients with ECRS is increasing, and the number of EOM cases may increase accordingly.^1^ ECRS and bronchial asthma were more frequently combined in patients with EOMs included in this study than in previous reports. Bronchial asthma was present in 100% of patients with EOM, and a high complication rate has been observed in previous reports.^7^, ^12^, ^15^, ^16^ Because bronchial asthma comorbidity is considered a risk factor for poor control of EOM, our EOM cases may have been more severe.^17^

EOM has been treated with tympanostomy and topical corticosteroids in the tympanic chamber and oral corticosteroids in severe cases; however, recently, biologics such as dupilumab, omalizumab, mepolizumab, and benralizumab have been reported to be effective^4^, ^8^, ^9^, ^18^, ^19^, ^20^, ^21^ on type 2 inflammation. Although there are currently no biologics covered by health insurance for EOM in Japan, EOM is associated with a high rate of ECRS and bronchial asthma, for which biologics are sometimes used. Severe cases of ECRS can be treated with dupilumab, and EOM may improve secondary to the administration of dupilumab for ECRS.^22^ The treatment of EOM is changing owing to the indirect influence of biologics, such as dupilumab. In fact, this review revealed that approximately half of EOM patients are currently receiving dupilumab for concomitant ECRS, and that EOM treatment has also changed significantly under the influence of dupilumab. In fact, this report revealed that approximately half of EOM patients are currently treated with dupilumab for concomitant ECRS, and that EOM treatment has also changed significantly due to dupilumab.

Patients with EOM administered dupilumab showed good improvement in EOM severity and CT score after 3-months of treatment. Additionally, EOM severity and temporal bone CT scores in patients treated with dupilumab were significantly better than those in patients with EOM treated without dupilumab. These results suggest that dupilumab is clinically effective in treating EOM. Furthermore, while some patients treated without dupilumab required regular or intermittent use of PSL, patients with EOM treated with dupilumab did not require PSL, suggesting that dupilumab also reduces steroid administration for the treatment of EOM. Similarly, Nakashima et al. reported that dupilumab for ECRS with EOM allowed completion of steroid therapy in 54-weeks.^22^

In 2020, dupilumab was covered by health insurance for CRSwNP in Japan and has shown good therapeutic outcomes in the treatment of ECRS, a typical disease of CRSwNP.^5^, ^6^ Biologics targeting IL-5 have also been shown to be effective against ECRS,^23^ and if they become available for administration in Japan, the treatment options for ECRS will increase. EOM, similar to ECRS, is closely related to type 2 inflammation,^1^ and biologics that are effective for ECRS are also expected to be effective for EOM. However, no biologics can be administered for EOM, and the treatment of EOM cases without concomitant ECRS or bronchial asthma, or without an indication for biologics for concomitant ECRS or bronchial asthma, must rely on the intratympanic or systemic administration of steroids.

Hearing loss greatly impairs the quality of life of patients with EOM. In our study, the air-conductive hearing of patients was significantly improved after dupilumab treatment. Additionally, air-conductive hearing was significantly better in patients treated with dupilumab than in those not treated with dupilumab. Type 2 inflammation of the middle ear in patients with EOMs may have been reduced by dupilumab, resulting in better air-conductive hearing. Prolonged middle ear inflammation due to EOM is associated with an elevated bone-conductive hearing threshold,^24^ and if better air-conductive hearing is maintained with dupilumab, progression to sensorineural hearing loss may be prevented. In fact, it has been reported that the administration of dupilumab to patients with EOM tends to prevent worsening of bone-conductive hearing thresholds.^9^ Therefore, dupilumab should ideally be administered to patients with EOM before sensorineural hearing loss progresses and becomes irreversible. Even in patients with irreversible sensorineural hearing loss, hearing improvement can be expected with cochlear implantation^25^, ^26^, ^27^; thus, it is highly important to control eosinophilic inflammation in the middle ear with dupilumab.

Dupilumab was administered to treat the ECRS. Dupilumab is indicated in patients with ECRS because of the poor condition of the ECRS; however, the severity of EOM at that time is not necessarily poor. The EOM severity score before treatment with dupilumab was 3.1 ± 0.9, which was poor compared to the severity score of 2.3 ± 2.3 at the time of evaluation of the 13 patients who were treated without dupilumab. Thus, the severity of EOM at the time dupilumab was administered to patients with ECRS was poor, and a significant improvement in the severity score 3-months after treatment with dupilumab was considered noteworthy.

One limitation of this study was its retrospective nature with a small number of cases. Further investigation involving a larger number of cases is warranted. Another limitation was that our study included suspected EOM cases that did not fulfill the diagnostic criteria for EOM. However, it has been reported that blood eosinophil concentration and eosinophil count are significantly higher in EOM compared to otitis media with effusion associated with granulomatosis with polyangiitis or primary ciliary dyskinesia. Furthermore, when a cutoff value of 6% for eosinophil concentration and 450 μL for eosinophil count was used, both the sensitivity and specificity for diagnosing EOM were found to be 100%.^28^ Our case also presented with middle ear effusion, and both the peripheral blood eosinophil percentage (14.2% ± 6.8%) and eosinophil count (978 ± 594) were elevated, strongly suggesting Eosinophilic Otitis Media (EOM) even in suspected cases.

## Conclusion

Our study demonstrated that dupilumab was administered to approximately half of patients with EOM, indicating that EOM treatment has undergone significant changes due to dupilumab administration in the ECRS. These results suggest that dupilumab is highly effective for the treatment of EOM.

## Funding

This study did not receive any specific grant from funding agencies in the public, commercial, or not-for-profit sectors.

## Declaration of competing interest

The authors declare no have conflicts of interest.
